# Enhancing Legged Robot Locomotion Through Smooth Transitions Using Spiking Central Pattern Generators

**DOI:** 10.3390/biomimetics10060381

**Published:** 2025-06-07

**Authors:** Horacio Rostro-Gonzalez, Erick I. Guerra-Hernandez, Patricia Batres-Mendoza, Andres A. Garcia-Granada, Miroslava Cano-Lara, Andres Espinal

**Affiliations:** 1 GEPI Research Group, IQS—School of Engineering, Ramon Llull University, Via Augusta 390, 08017 Barcelona, Spain; 2 Department of Electronics Engineering, DICIS—University of Guanajuato, Carretera Salamanca—Valle de Santiago km 3.5 + 1.8 kms, Salamanca 36885, Mexico; andres.garcia@iqs.url.edu; 3 Laboratory of Artificial Intelligence, Robotics and Control, Faculty of Biological Systems and Technological Innovations, Benito Juárez Autonomous University of Oaxaca, Oaxaca 68120, Mexico; ghernandez.cat@uabjo.mx (E.I.G.-H.); bmendoza.cat@uabjo.mx (P.B.-M.); 4 Department of Mechatronics, TecNM/ITS Irapuato, Irapuato 36821, Mexico; miroslava.cl@irapuato.tecnm.mx; 5 Department of Organizational Studies, University of Guanajuato, Fraccionamiento 1, Col. El Establo S/N, Marfil 36250, Mexico; aespinal@ugto.mx

**Keywords:** spiking neurons, Central Pattern Generators, legged robots, lomocotion, gait transitions

## Abstract

In this work, we propose the integration of a mechanism to enable smooth transitions between different locomotion patterns in a hexapod robot. Specifically, we utilize a spiking neural network (SNN) functioning as a Central Pattern Generator (CPG) to generate three distinct locomotion patterns, or gaits: walk, jog, and run. This network produces coordinated spike trains, mimicking those generated in the brain, which are translated into synchronized robot movements via PWM signals. Subsequently, these spike trains are compared using a similarity metric known as SPIKE-synchronization to identify the optimal point for transitioning from one gait to another. This approach aims to achieve three main objectives: first, to maintain the robot’s balance during transitions; second, to ensure that gait transitions are almost imperceptible; and third, to improve energy efficiency by reducing abrupt changes in the robot’s actuators (servomotors). To validate our proposal, we incorporated FSR sensors on the robot’s legs to detect the rigidity of the terrain it navigates. Based on the terrain’s rigidity, the robot dynamically transitions between gaits. The system was tested in real time on a physical hexapod robot across four different types of terrain. Although the method was validated exclusively on a hexapod robot, it can be extended to any legged robot.

## 1. Introduction

Legged robots have emerged as a class of robots capable of navigating diverse and challenging environments where wheeled or tracked systems often fall short [1,2]. From uneven terrains to cluttered indoor spaces, legged robots provide superior mobility by mimicking the movement patterns of biological organisms [3]. Their ability to traverse complex surfaces, such as rocky landscapes, stairs, or loose soil, makes them ideal for applications in search and rescue missions, space exploration, and industrial inspection [4]. However, one of the main challenges in developing robust and efficient legged robots lies in achieving smooth transitions between various gait patterns and operational conditions [5,6]. Abrupt shifts in motion, whether due to changes in speed, direction, or terrain type can lead to instability, energy inefficiency, and mechanical stress [7,8].

Legged robots rely on a variety of gaits, which are coordinated patterns of limb movement that allow them to walk, run, jump, or climb [9]. These gaits are typically selected based on environmental conditions and the desired speed or energy consumption of the robot [10]. For instance, walking gaits are preferred at low speeds on rough terrain for stability, while running gaits are more effective for high-speed movement on flat surfaces [11,12]. However, the transition between these gaits, especially in dynamic or unpredictable environments remains a challenge. Poorly timed or abrupt transitions can compromise balance, increase energy consumption, and cause mechanical failures [10]. Achieving smooth, adaptive transitions, such as from walking to running or during surface changes, is thus vital for maintaining the robot’s balance, stability, and efficiency [10].

In biological systems, animals transition effortlessly between gaits, adjusting their movement patterns based on terrain and speed [13]. This is enabled by Central Pattern Generators (CPGs), neural circuits capable of producing rhythmic outputs without requiring continuous sensory feedback [14,15]. These circuits are found in the spinal cords of vertebrates and control repetitive locomotion patterns such as walking, swimming, or flying [16]. Inspired by this biological mechanism, researchers have applied CPG-based control systems in robotics to generate smooth and adaptive limb coordination [5]. The use of spiking neurons within CPGs further enhances the biological realism and timing precision of these control systems [17], enabling robots to better emulate the gait modulation observed in animals [18].

Spiking neural networks (SNNs) have demonstrated great potential in bio-inspired robotic control due to their event-based, low-latency communication properties [19,20,21]. However, most existing studies using SNNs for locomotion rely on large-scale networks or are restricted to simulation environments, requiring high-performance computing platforms for real-time operation [22,23,24]. These factors limit their use in practical applications, especially on low-cost, resource-constrained robotic systems.

In this paper, we address this gap by introducing a novel, fully bio-inspired spiking CPG controller composed of only 12 neurons capable of producing distinct gait patterns and achieving smooth transitions between them. Gait changes are autonomously triggered based on terrain feedback, using a Force-Sensitive Resistor (FSR) sensor integrated into the hexapod’s leg. Crucially, this minimalist architecture enables real-time performance on low-power microcontrollers such as an Arduino board, demonstrating a level of computational and energy efficiency not seen in other SNN-based approaches.

Through simulation and extensive real-world experiments, we demonstrate that our system achieves nearly imperceptible gait transitions, with average stepping time errors below 5 milliseconds, while operating on minimal hardware. These results highlight a path forward for efficient, adaptive locomotion in field-ready legged robots.

The rest of this paper is organized as follows: Section 2 describes the robot architecture, the Spiking Central Pattern Generator (SCPG), the SPIKE-synchronization metric, and the hardware implementation. In Section 3, we present the performance of our approach under various conditions, along with a detailed performance analysis. Section 4 provides a discussion of the results, and finally, Section 5 concludes the paper.

### Problem Statement

**Figure 1:**
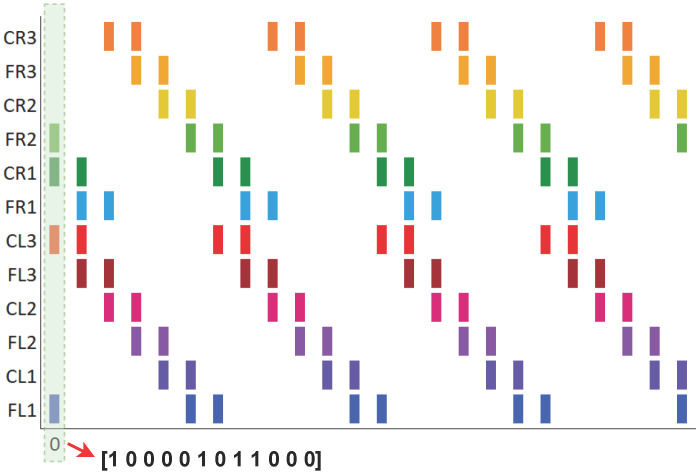
Locomotion pattern “walk”, where each colored line on the graph represents the activation of a servo at a given time. The vertical axis labels the servos (or neurons), with C and F indicating the coxa and femur, respectively, and R and L denoting the right and left sides.

In [25], a spiking neural network was proposed to reproduce three biologically inspired gait patterns—walk, jog, and run—observed in stick insects, aimed at controlling the locomotion of legged robots. The transition between these gait patterns was managed by initializing a vector that defined the activation of each servomotor in the robot at time zero. In other words, for each gait pattern, there was an initial vector of pulse values (0 or 1), and from this vector, the spiking neural network, as described by Equations (1) and (2), generated the periodic activation of the neurons (or servos) during the execution of the gait sequence. An example of these vectors is shown in Figure 1. The issue with this approach is that the transition between patterns did not account for the current running time, leading to abrupt shifts that could affect or even damage the servos. This lack of proper synchronization increased the risk of the robot malfunctioning or crashing due to poor coordination between the servos during the transition.

To address this issue, the proposed solution in this research is to enable smooth transitions between gaits by monitoring the activation pulses (spikes) over time and selecting the optimal moment for transitioning. To achieve this, we use an event-based metric called SPIKE-synchronization, which will be explained in detail later.

When the network detects a change in $I^{ext}$ (see Equation (1)) triggered by a sensor input, e.g., an FSR sensor, such as when the robot encounters an obstacle or when the terrain type changes, the system will evaluate the SPIKE-synchronization metric to determine the best timing for the gait transition. This ensures a smoother, more coordinated change, reducing the risk of servo damage or robot malfunction.

## 2. Materials and Methods

### 2.1. The Robot

**Figure 2:**
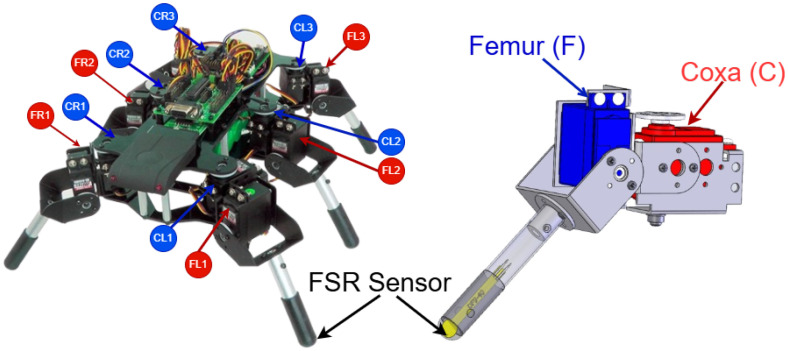
Robot platform overview. The left image shows the hexapod robot structure, with the servomotors controlling the coxa (C) and femur (F) joints highlighted in red and blue, respectively. The labels R and L correspond to the right and left legs. The right image shows a detailed view of a robot limb, showing the femur (F) and coxa (C) servomotors. The coxa joint enables forward and backward movement of the leg, while the femur joint controls the lifting and lowering of the limb. An FSR (Force-Sensitive Resistor) sensor is attached to the distal end of one of the robot’s legs, indicated in yellow.

In this work, we consider the hexapod robot shown in Figure 2, which consists of 12 servomotors, 2 per leg, responsible for controlling the coxa and femur. The control system includes an Arduino board and a servo controller capable of sending signals to all 12 servomotors simultaneously. Originally, this robot did not come equipped with any processing system, allowing for the integration of any processing unit. For this project, we selected an Arduino board since the neural network we developed comprises only 12 neurons. Additionally, force sensors (FSRs) have been installed at the base of the robot’s legs to enable it to detect the type of terrain it navigates (see Figure 2). These sensors allow the robot to assess terrain hardness and adjust its gait accordingly.

The following sections detail how the robot’s locomotion gaits are generated and how it interacts with its environment using the FSR sensors.

### 2.2. Spiking Central Pattern Generators

**Figure 3:**
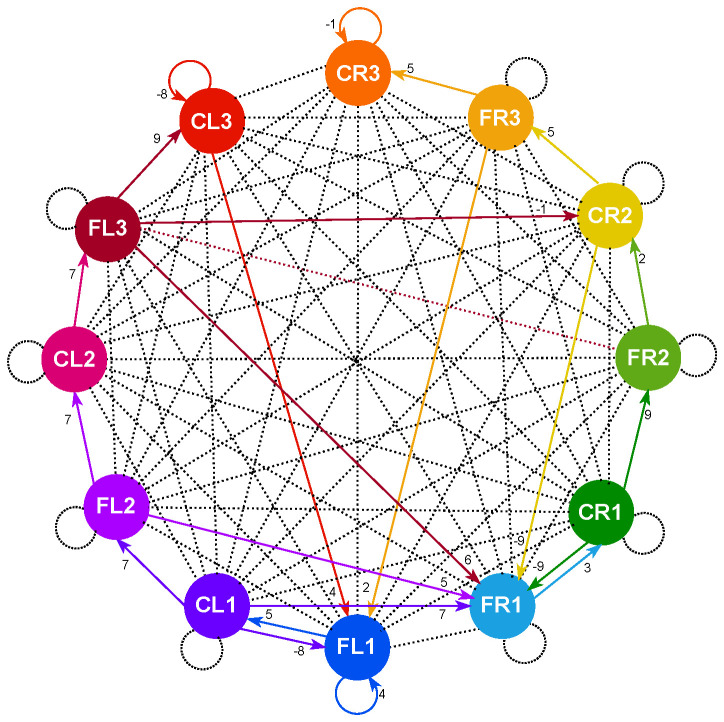
Topology of the Spiking Central Pattern Generator.

The robot’s locomotion is achieved through the synchronized activation of its six legs, each powered by two servomotors corresponding to the coxa and femur (see Figure 2), totaling 12 servomotors to control. This control is performed by a bio-inspired mechanism based on a Spiking Neural Network (SNN) (see Figure 3), which acts as a Central Pattern Generator [17], where each neuron corresponds to a servo. The spiking activity generated by the SNN controls the activation and deactivation of the servos, and is modeled by the following equations:

(1) $$V_{i}\left[k\right]=\begin{array}{c} \gamma V_{i}[k-1](1-Z[K-1]) \end{array}+\begin{array}{c} \sum_{j=1}^{N}W_{ij}Z_{j}\left[k-1\right] \end{array}+\begin{array}{c} I_{i}^{ext} \end{array}$$

and

(2) $$Z_{i}=\left\{\begin{array}{cc} 1 & if\;V\geq \theta \\ 0 & otherwise \end{array}\right.$$

Equation (1) presents a discretized representation of the electrical activity in the brain, resulting from the interactions and information processing between neurons [26]. This equation is a simplified version of the widely recognized generalized Leaky Integrate-and-Fire (LIF) model [27].

Specifically, Equation (1) describes the behavior of the neuron’s membrane voltage (*V*). This equation is composed of three key components: the first (in purple) represents the neuron’s internal state, the second (in green) accounts for synaptic activity with other neurons, and the third (in blue) represents an external current (sensory information). In our case, this external current is responsible for triggering the gait transition based on the data collected by force sensors placed on the legs.

In this equation, the value of *N* is 12, which corresponds to the 6 coxae and 6 femurs of the robot. In this context, this equation describes the membrane voltage generated for the synchronous interaction of these 12 elements. The value of γ is a discharge factor for the neuron, which penalizes the membrane voltage if the neuron has not fired. The firing of the neuron is described by Equation (2), which produces a binary output: 1 when the neuron reaches a threshold and 0 when it does not. In the latter case, the gamma factor comes into play, affecting the voltage. Its value ranges from [0,1). A value close to 0 indicates a strong impact on the membrane potential, while a value close to 1 results in minimal penalization. In our case, we considered γ=0.5. This value of γ was primarily chosen with digital hardware implementations in mind, as being a power of 2 (i.e., 0.5 = $2^{-1}$) allows for efficient implementation. However, this value can be any number between 0 and 1.

The value of *Z* in Equation (2) subsequently controls the activation or deactivation of a Pulse Width Modulation (PWM) signal, which drives the robot’s movement. This process is explained in Section 2.4.

(3) $$\begin{array}{ccc} W & = & \left[\begin{array}{cccccccccccc} 0 & 8 & 0 & 0 & 0 & 0 & 0 & 0 & 0 & 0 & 0 & 0\\ -7 & 0 & 4 & 2 & -2 & 0 & 1 & 2 & 0 & 0 & 2 & 0\\ -9 & -5 & 0 & 8 & 4 & 1 & 0 & -2 & 4 & 0 & 0 & 0\\ 0 & 0 & 0 & 0 & 8 & 0 & 0 & 0 & 0 & 0 & 0 & 0\\ 0 & 0 & 0 & 0 & 0 & 5 & 0 & 0 & 0 & 0 & 0 & 0\\ 6 & 0 & 0 & 0 & -9 & 0 & 0 & 0 & 3 & 0 & 3 & -1\\ 0 & 0 & 0 & 0 & 0 & 0 & 0 & 5 & 0 & 0 & 0 & 0\\ 0 & 0 & 0 & 0 & 0 & 0 & 5 & 0 & 4 & 0 & -2 & 0\\ 0 & 0 & 0 & 0 & 0 & 0 & 0 & 0 & 0 & 8 & 0 & 0\\ 0 & 0 & 0 & 0 & 0 & 0 & 0 & 0 & 0 & 0 & 9 & 0\\ 0 & -4 & 0 & 0 & 0 & -3 & 0 & -2 & 0 & 0 & 3 & 9\\ 0 & 1 & 0 & -3 & 8 & -1 & 5 & 0 & -4 & 0 & -4 & 0 \end{array}\right] \end{array}$$

**Figure 4:**
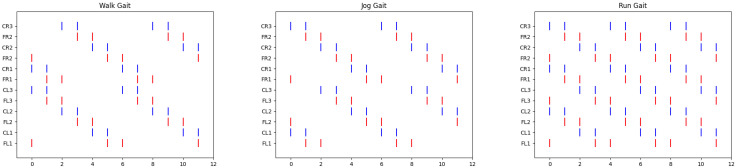
Gait locomotion patterns for different movement speeds: walking (**left**), jogging (**middle**), and running (**right**). Colored lines indicate servo activations (red for the coxae and blue for the femurs).

Figure 3 illustrates the schematic topology of the spiking neural network designed for generating three distinct gaits: walk, jog, and run. This topology was developed using a grammar evolutionary algorithm, as detailed in [28]. The synaptic weight matrix employed for creating these three locomotion patterns is given in Equation (3). An important aspect during the estimation of synaptic weights by the algorithm is that they were restricted to integer values only to further reduce the computational cost of implementing the system in hardware, as using only integer weights cuts in half the number of bits required compared to using real values for hardware representation. From this topology, we successfully generated gait patterns, as demonstrated in Figure 4, corresponding to each of the three gaits.

A notable aspect of the patterns observed in Figure 4 is their replication of the gait patterns found in insects, namely tripod and tetrapod gait patterns [29]. According to previous studies, the tripod gait facilitates faster locomotion, as evidenced in Figure 4 (see [30]). In contrast, the tetrapod gait is better suited for medium- and low-speed locomotion.

### 2.3. SPIKE-Synchronization

Once the different gait patterns are generated, the next step is to evaluate the hexapod robot’s ability to transition between them. For this, we use a mechanism known as SPIKE-synchronization, which allows us to assess the current gait state and identify the closest match to the desired target gait. This method applies to transitions aimed at both increasing and decreasing walking speed.

**Figure 5:**
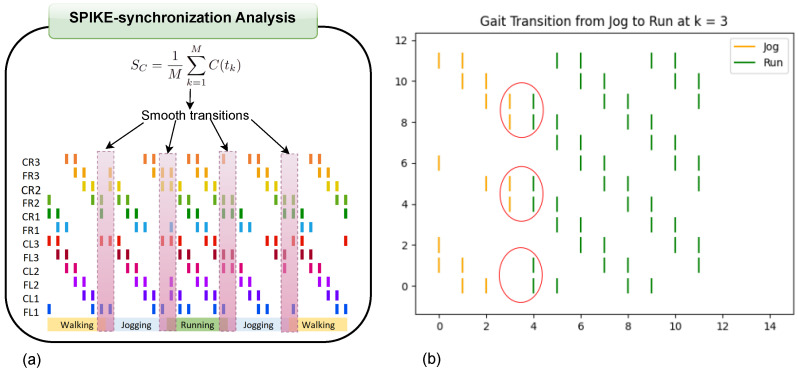
SPIKE- synchronization: (**a**) general scheme and (**b**) example of transition from jogging to running at time k=3.

The SPIKE-synchronization is a parameter- and scale-free metric that quantifies the degree of synchrony between events, such as neuronal spikes (see Figure 5a), providing insights into the temporal alignment of these occurrences [31]. This similarity measure produces high values for two similar spike trains; in other words, it reaches a value of one if and only if the two spike trains consist solely of pairs of coincident spikes and a value of zero for spike trains without any coincidences. Let $t_{i}^{\left(1\right)},i=1,\ldots ,M_{1}$, and $t_{j}^{\left(2\right)},j=1,\ldots ,M_{2}$, (where $M_{n}$ is the number of spikes in spike train *n*, with n=1,2) be the sequences of discrete spike times. As a starting point, SPIKE-synchronization requires the definition of coincidence detection, which commonly uses a coincidence window $\tau_{ij}^{\left(1,2\right)}$ (see Equation (4)), referring to the time lag within which two spikes from $t_{i}^{\left(1\right)}$ and $t_{j}^{\left(2\right)}$ are considered coincident.

(4) $$\tau_{ij}^{\left(1,2\right)}=\min\left\{t_{i+1}^{\left(1\right)}-t_{i}^{\left(1\right)},t_{i}^{\left(1\right)}-t_{i-1}^{\left(1\right)},t_{j+1}^{\left(1\right)}-t_{j}^{\left(2\right)},t_{j}^{\left(2\right)}-t_{j-1}^{\left(2\right)}\right\}/2$$

The coincidence specification can be measured using the coincidence indicator in Equation (5), which assigns a value of one to each spike if it is part of a coincidence or zero if it is not; the same rule applies to $C_{j}^{\left(2\right)}$.

(5) $$C_{i}^{1}=\left\{\begin{array}{cc} 1 & \mathrm{if}\;\min_{j}\left(\right|t_{i}^{\left(1\right)}-t_{j}^{\left(2\right)}\left|\right)<\tau_{ij}^{\left(1,2\right)}\\ 0 & \mathrm{otherwise} \end{array}\right.$$

Once the coincidence indicator for each individual spike of the two spike trains is defined, the spike trains and their coincidence indicators are aggregated in order to obtain a combined similarity profile by establishing an overall spike index *k*, producing a consolidated set of coincidence indicators $C_{k}$ in which each coincidence produces a pair of consecutive ones. The SPIKE-synchronization in Equation (6) is defined as the average value of the profile $C(t_{k})$ with $C\left(t_{k}\right)=C\left(k\right)$.

(6) $$S_{C}=\frac{1}{M}\sum_{k=1}^{M}C\left(t_{k}\right)$$

where $M=M_{1}+M_{2}$ is the total number of spikes in the combined spike trains. $S_{c}$ can be interpreted as a measure of the fraction of all coincident spikes in the two spike trains; thus, since the metric is defined as the proportion between the number of spike coincidences and the total number of spikes, it is a dimensionless quantity.

Figure 5b illustrates a transition from jogging to running at time step k=3. At this moment, the SPIKE-synchronization metric reaches the best match for a smoother transition.

### 2.4. Hardware Implementation

The Spiking Neural Network used in this study is relatively small, comprising only 12 neurons with a maximum connectivity of 144 when fully connected. However, this is not the case here, as several connection weights are zero (see Equation (3)). For this reason, we opted for a low-cost implementation using an Arduino UNO board. In the future, a neuromorphic system such as SpiNNaker [32] or Loihi [33] could be employed to incorporate a bio-inspired hardware component, as these architectures allow for the direct integration of spiking neuron models.

The 144 possible connections are represented as real (float) values, with each occupying 4 bytes, resulting in a total memory usage of 576 bytes. Considering the Arduino UNO has 32 kB of memory, this implementation utilizes only a small fraction of its capacity.

The other elements in Equation (1) consume minimal resources. The membrane potential requires a real-type variable as well, meaning a total of 12 values need to be stored at each time step, requiring an additional 48 bytes. The parameter γ is a constant value that consumes 4 more bytes. Lastly, *Z* is a vector of 12 boolean elements.

In total, the memory usage is around 700 bytes, which is approximately 2% of Arduino’s total available memory.

**Figure 6:**
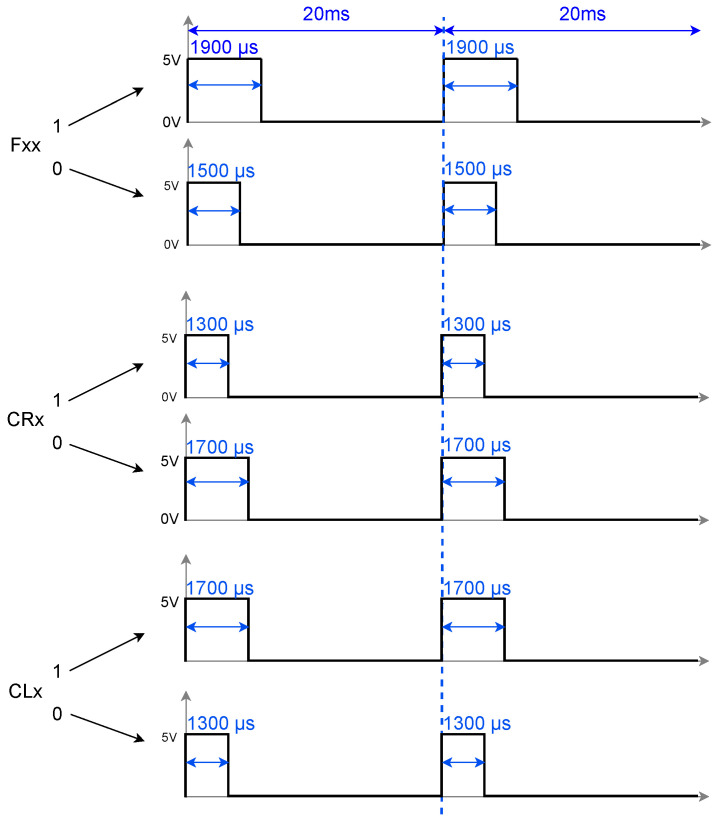
Spikes to PWM.

On the implementation itself, Equations (1) and (2) have been fully mapped in Arduino, generating a topology as shown in Figure 3, which is in charge of controlling each of the servo motors in the robot (see Algorithm 1). In principle, each of these neurons will be emitting binary pulses, which will represent activation or non-activation of the servo motors in a synchronous manner. However, for this to represent motion in the robot, a PWM signal as shown in Figure 6 must be used.
**Algorithm 1** Spiking Neural Network (SNN) update algorithm  1:**function** SNN(*V*, *Z*, *W*, $I^{\mathrm{ext}}$, γ, θ, *N*)  2:      **for** i=1 to *N* **do**  3:            sum = 0  4:            **for** j=1 to *N* **do**  5:                  sum=sum+W[i,j]·Z[i]  6:            **end for**  7:            $V\left[i\right]=\gamma \cdot V\left[i\right]\cdot \left(1-Z\left[i\right]\right)+sum+I^{\mathrm{ext}}\left[i\right]$  8:            **if** V[i]≥θ **then**  9:                  Z[i]=110:            **else**11:                  Z[i]=012:            **end if**13:      **end for**14:      **return** *V*, *Z*15:**end function**

The conversion of spikes to PWM signals shown in Figure 6 allows the neuronal activity to be translated into control commands for the servomotors. In this case, a direct relationship is established between the firing frequency of the neuron and the duty cycle of the PWM signal. For the femur, when no spike is emitted by the neuron, a PWM signal with a duty cycle of 1500 μs is generated. However, when a spike occurs, the duty cycle increases to 1900 μs. In the right coxae, a similar behavior is observed but with inverted duty cycle values: 1700 μs when the neuron is at rest and 1300 μs during the emission of a spike. On the other hand, in the left coxae, this relationship is inverted, due to the fact that the servomotors are rotated 180° with respect to the right coxae, that is, the duty cycle is lower (1300 μs) when the neuron does not fire and higher (1700 μs) during the emission of a spike.

## 3. Results

**Figure 7:**
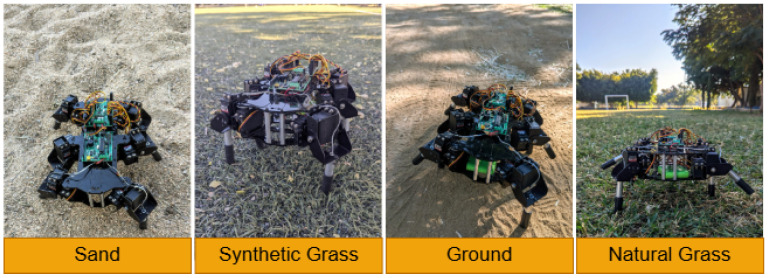
Hexapod robot interaction on regular and irregular surfaces.

To validate our method, we tested our hexapod robot’s navigation across four different surfaces—sand, ground, synthetic grass, and natural grass—as shown in Figure 7. Equipped with Force-Sensitive Resistor (FSR) sensors, the robot is capable of detecting variations in terrain based on changes in resistance measured by the sensors. Table 1 summarizes the mean resistance values observed for each type of terrain. These sensor readings serve as the input for triggering gait transitions in the robot. By identifying specific resistance thresholds associated with each surface, the robot can adapt its gait dynamically.

SPIKE-synchronization analysis for the different possible gait transitions is presented in Table 2, Table 3, Table 4, Table 5 and Table 6; the rows represent the different patterns at time *k* for the ongoing gait, and the columns correspond to the possible patterns of the transitioning gait, except for the last column, which indicates the selected time of the transitioning gait that best matches the ongoing gait at time *k*. Since a SPIKE-synchronization value closer to 1 indicates greater similarity, at each time step *k*, all potential states of the transitioning gait are evaluated with respect to the ongoing gait in order to identify the one that best matches, thus enabling a smooth transition; in cases where multiple patterns of the transitioning gait reach the highest value, the first one is selected for pairing.

**Figure 8:**
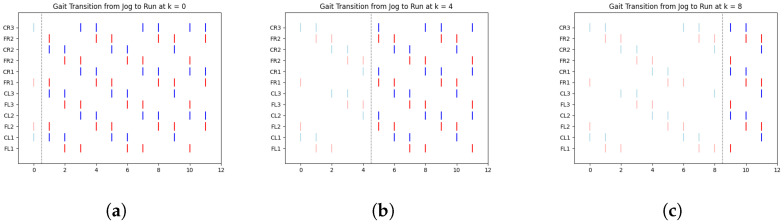
Gait transition from jog to run at (**a**) early, (**b**) middle, and (**c**) late simulation time. These plots present representative examples of neural spike activity during transitions. Red and blue traces correspond to activity in the coxa and femur joints, respectively, reflecting the activation or deactivation of the associated motors.

In Figure 8, we illustrate a clear example of the transition from the ongoing gait (jog) to the transitioning gait (run) at early, middle, and late simulation times. Each figure includes a vertical dashed line marking the exact moment of transition from one gait to another.

**Figure A1:**
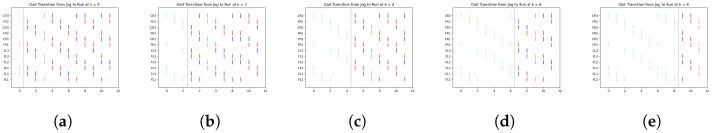
Gait transition from jog to run at different time steps. These plots present representative examples of neural spike activity during transitions. Red and blue traces correspond to activity in the coxa and femur joints, respectively, reflecting the activation or deactivation of the associated motors.

**Figure A2:**
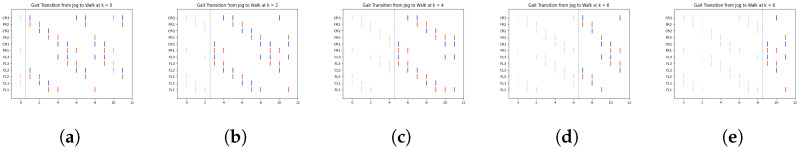
Gait transition from jog to walk at different time steps. These plots present representative examples of neural spike activity during transitions. Red and blue traces correspond to activity in the coxa and femur joints, respectively, reflecting the activation or deactivation of the associated motors.

**Figure A3:**
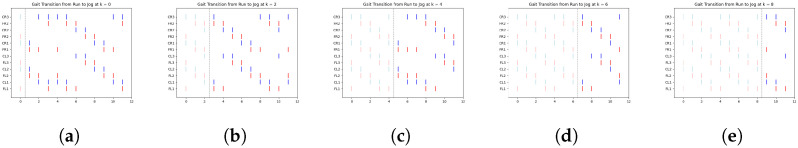
Gait transition from run to jog at different time steps. These plots present representative examples of neural spike activity during transitions. Red and blue traces correspond to activity in the coxa and femur joints, respectively, reflecting the activation or deactivation of the associated motors.

**Figure A4:**
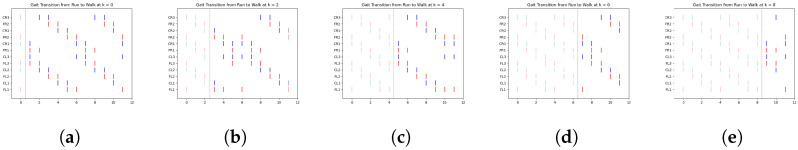
Gait transition from run to walk at different time steps. These plots present representative examples of neural spike activity during transitions. Red and blue traces correspond to activity in the coxa and femur joints, respectively, reflecting the activation or deactivation of the associated motors.

**Figure A5:**
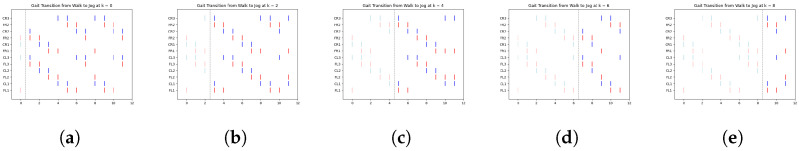
Gait transition from walk to jog at different time steps. These plots present representative examples of neural spike activity during transitions. Red and blue traces correspond to activity in the coxa and femur joints, respectively, reflecting the activation or deactivation of the associated motors.

**Figure A6:**
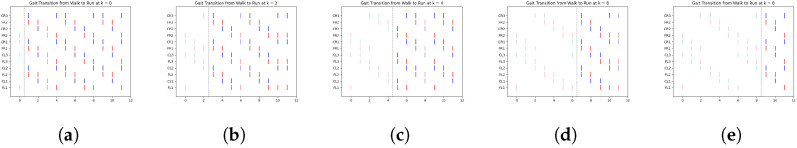
Gait transition from walk to run at different time steps. These plots present representative examples of neural spike activity during transitions. Red and blue traces correspond to activity in the coxa and femur joints, respectively, reflecting the activation or deactivation of the associated motors.

According to Table 4, Figure 8a shows the jog gait at simulation time 0, which transitions to the run gait pattern at simulation time 1. Similarly, Figure 8b shows the jog gait at simulation time 4, transitioning to the run gait pattern at simulation time 5. Finally, Figure 8c shows the jog gait at simulation time 8, transitioning to the run gait pattern at simulation time 9. The complete set of gait transitions is shown in Appendix A, in Figure A1, Figure A2, Figure A3, Figure A4, Figure A5 and Figure A6. A total of 60 experiments were conducted, with 10 trials for each transition type. However, for clarity and better visualization, only five trials per transition are presented.

The dashed line represents the point at which the FSR sensor detected a change in terrain, prompting a gait transition. The spikes are color-coded: red represents the activation of the motor controlling the coxa, while blue corresponds to the femur.

Although the simulation time shown in Appendix A Figure A1, Figure A2, Figure A3, Figure A4, Figure A5 and Figure A6 extends up to 12 time steps, these Table 2, Table 3, Table 4, Table 5 and Table 6 display data only up to time step 5. This is due to the periodic nature of the gaits, where the pattern begins to repeat after this point. By focusing on the initial time steps, we can better analyze the effectiveness of the transitions without redundancy.

One particularly interesting observation in these graphs is the shift in balance structure when transitioning between jog/walk and run, or vice versa. During these transitions, the robot switches between tripod and tetrapod balance structures, as described in [29]. This shift highlights the adaptability of the system to changes in terrain and gait demands, demonstrating the robustness of the proposed approach.

### Performance Evaluation

To quantitatively evaluate the performance of our approach, Table 7 presents a comparison of the stepping times (in microseconds) during gait transitions in the hexapod robot. The table includes the expected values, the experimentally measured values, and the corresponding errors for each transition. The results show that the average errors range from 3.3 to 4.8 milliseconds, with an overall mean error of 4.043 milliseconds.

Additionally, Table 8 presents the energy consumption, measured in terms of the current, voltage, and power of the electronics and servomotors of the hexapod robot under different gait patterns: running and walking/jogging. The data reflect the energy demands during stable operating conditions at 6 V, and the power was calculated using the equation P=VI, where *I* was measured during execution time.

## 4. Discussion

While biological inspiration has long guided research in robotic locomotion, the use of spiking neural networks (SNNs) in this field remains relatively limited. Most studies in gait generation rely on traditional control systems or oscillators, with only a few exploring the unique potential of spike-based, event-driven computation.

Among the existing SNN-based approaches, many employ large and complex networks to generate rhythmic locomotion patterns. For instance, systems such as those implemented on neuromorphic platforms like SpiNNaker or Loihi typically involve hundreds or even thousands of spiking neurons to model Central Pattern Generators (CPGs) or sensory–motor integration modules. Although these implementations have demonstrated biological plausibility and functional control, they come at the cost of high computational complexity and often require specialized hardware.

In contrast, our proposal introduces a fully bio-inspired architecture that operates with only 12 spiking neurons, which is significantly fewer than what has been reported in the literature. This minimalist design is capable of generating and transitioning between three distinct gait patterns—walk, jog, and run—without relying on complex training procedures or extensive synaptic configurations.

A key innovation in our system is the event-based transition mechanism, which is triggered by sensory input from a Force-Sensitive Resistor (FSR). This sensor detects terrain rigidity, allowing the robot to adapt its locomotion speed accordingly. To the best of our knowledge, no previous work has demonstrated such gait transitions driven by event-based sensory mechanisms in SNN-controlled robots.

Furthermore, the low computational demands of our network allow the entire system to run on a low-cost microcontroller platform, such as Arduino, as opposed to power-hungry processors or external compute units. This not only reduces cost and system complexity but also contributes to greater energy efficiency—an essential feature for autonomous, battery-powered robots.

As a future research direction, we envision extending this bio-inspired architecture by incorporating neuromorphic sensors, such as event-based vision sensors (e.g., Dynamic Vision Sensors (DVSs)) or tactile neuromorphic interfaces. These sensors would provide spiking input natively, enabling more seamless and biologically plausible integration with the SNN controller while also potentially allowing the robot to adapt to more complex and dynamic environments with enhanced sensory resolution and lower latency.

## 5. Conclusions

The aim of our work is to reduce the gap between basic research, as exemplified in our previous studies, where we successfully designed a unique Spiking Central Pattern Generator capable of generating multiple gaits and applied research, which is the current focus of our efforts to extend the capabilities of our proposal. In this regard, we explored the integration of a transition mechanism between gaits in the locomotion of a hexapod robot, aiming to make these transitions as smooth as possible. To achieve this, we utilized an event-based metric that measures the degree of similarity between two spike trains (neural activations). This metric was incorporated because the robot’s locomotion is controlled by a spiking neural network (SNN), which generates three gait patterns (walk, jog, and run) based on events, or spikes. These patterns are produced by a single network capable of transitioning from one gait to another, with the transitions triggered by the FSR readings based on the terrain conditions.

A critical aspect of our approach, as observed in real-world experiments showcasing the robot’s gait transitions, is the deliberate modulation of locomotion speed in response to the terrain. Specifically, the robot adjusts its velocity, slowing down or speeding up, based on the rigidity of the surface, as measured by an integrated Force-Sensitive Resistor (FSR) sensor. This adaptive behavior is fundamental to the system, allowing the robot to maintain stability and efficiency when navigating different environments. The observed delay during gait transitions, which reflects the time needed for the robot to adjust its speed, is quantitatively captured and discussed through the stepping time error presented in Table 7. Notably, the transition error is nearly imperceptible, with a maximum average stepping time error of 0.004781 s in the worst-case scenario.

We conducted numerous experiments on a real hexapod robotic platform with a low-cost processing unit, namely Arduino. While not every trial resulted in a perfectly smooth transition, the majority achieved transitions that were nearly imperceptible, highlighting the potential of the proposed approach to improve robotic locomotion performance.

Although experimentation was carried out on a six-legged platform, the methodology is extensible to robots with varying numbers of limbs. Furthermore, integrating neuromorphic platforms such as SpiNNaker or Loihi could enhance the system, advancing toward a fully bio-inspired robotic locomotion framework. This paves the way for more energy-efficient and adaptable robots capable of navigating complex environments with improved robustness and flexibility.

## Figures and Tables

**Table 1:** Mean of 10 FSR readings across the four different surfaces.

	Surface			
	Sand	Ground	Synthetic Grass	Natural Grass
MEAN	14.77 KΩ	6.55 KΩ	9.29 KΩ	33.83 KΩ

**Table 2:** SPIKE-synchronization values for times of transition from walk (row) to jog (column) and the specific time to perform the transition. The same distance values are generated for the opposite case (from jog to walk). The values in bold indicate the optimal transition values according to the SPIKE-distance metric.

k	0	1	2	3	4	5	Nearest Time Selected
0	0.00	0.25	**0.50**	0.50	0.50	0.25	2
1	0.25	0.00	0.25	**0.50**	0.50	0.50	3
2	**0.50**	0.25	0.00	0.25	0.50	0.50	0
3	**0.50**	0.50	0.25	0.00	0.25	0.50	0
4	**0.50**	0.50	0.50	0.25	0.00	0.25	0
5	0.25	**0.50**	0.50	0.50	0.25	0.00	1

**Table 3:** SPIKE-synchronization values for times of transition from walk (row) to run (column) and the specific time to perform the transition. The values in bold indicate the optimal transition values according to the SPIKE-distance metric.

k	0	1	2	3	Nearest Time Selected
0	**0.60**	0.20	0.20	0.60	0
1	**0.40**	0.40	0.40	0.40	0
2	**0.60**	0.60	0.20	0.20	0
3	0.40	**0.80**	0.40	0.00	1
4	0.00	0.40	**0.80**	0.40	2
5	0.40	0.00	0.40	**0.80**	3

**Table 4:** SPIKE-synchronization values for times of transition from jog (row) to run (column) and the specific time to perform the transition. The values in bold indicate the optimal transition values according to the SPIKE-distance metric.

k	0	1	2	3	Nearest Time Selected
0	0.20	**0.60**	0.60	0.20	1
1	**0.40**	0.40	0.40	0.40	0
2	0.20	0.20	**0.60**	0.60	2
3	0.40	0.00	0.40	**0.80**	3
4	**0.80**	0.40	0.00	0.40	0
5	0.40	**0.80**	0.40	0.00	1

**Table 5:** SPIKE-synchronization values for times of transition from run (row) to walk (column) and the specific time to perform the transition. The values in bold indicate the optimal transition values according to the SPIKE-distance metric.

k	0	1	2	3	4	5	Nearest Time Selected
0	**0.60**	0.40	0.60	0.40	0.00	0.40	0
1	0.20	0.40	0.60	**0.80**	0.40	0.00	3
2	0.20	0.40	0.20	0.40	**0.80**	0.40	4
3	0.60	0.40	0.20	0.00	0.40	**0.80**	5

**Table 6:** SPIKE-synchronization values for times of transition from run (row) to jog (column) and the specific time to perform the transition. The values in bold indicate the optimal transition values according to the SPIKE-distance metric.

k	0	1	2	3	4	5	Nearest Time Selected
0	0.20	0.40	0.20	0.40	**0.80**	0.40	4
1	0.60	0.40	0.20	0.00	0.40	**0.80**	5
2	**0.60**	0.40	0.60	0.40	0.00	0.40	0
3	0.20	0.40	0.60	**0.80**	0.40	0.00	3

**Table 7:** Mean stepping time error between the robot’s legs during each transition under different conditions. The values reflect the temporal synchronization performance of the gait generated by the spiking neural network.

Stepping Time (μs)			
Transition	Expected	Measured	Error
Jogging to Walking	1,644,939	1,648,263	3324
Running to Walking	1,644,939	1,649,566	4627
Walking to Jogging	1,244,965	1,248,307	3342
Running to Jogging	1,244,965	1,249,542	4577
Jogging to Running	1,246,622	1,251,403	4781
Walking to Running	1,246,622	1,250,229	3607
mean			4043

**Table 8:** Energy consumption (current, voltage, and power) of the hexapod robot’s electronics and servomotors under different gait patterns: running and walking/jogging.

Gait	I (mA)	V (V)	P (W)
Running	227	6	13.62
Walking/Jogging	191	6	11.46

## Data Availability

No new data were generated for this research; however, real-world tests conducted on the robotic platform can be found at the following link: https://github.com/BioInspiredLab-UGTO/Smooth-CPG (accessed on 3 June 2025).
